# Selenium Nanoparticles Improved Intestinal Health Through Modulation of the NLRP3 Signaling Pathway

**DOI:** 10.3389/fnut.2022.907386

**Published:** 2022-07-05

**Authors:** Yanhong Chen, Wei Wu, Huajin Zhou, Xingbo Liu, Shu Li, Yanbing Guo, Yuxin Li, Yongqiang Wang, Jianmin Yuan

**Affiliations:** ^1^State Key Laboratory of Animal Nutrition, College of Animal Science and Technology, China Agricultural University, Beijing, China; ^2^College of Resources and Environmental Sciences, China Agricultural University, Beijing, China; ^3^Beijing Wahmix Bio-Technology Co., Ltd., Tangshan, China; ^4^Microbiology and Immunology Department of Preventive Veterinary Medicine, College of Veterinary Medicine, China Agricultural University, Beijing, China

**Keywords:** selenium nanoparticles (SeNPs), goblet cells, NLRP3, broiler, intestine

## Abstract

Selenium nanoparticles (SeNPs) play important roles in promoting animal health, however, their impact on intestinal health remains elusive. This study was intended to evaluate the effects of different doses of SeNPs on the intestinal health, especially the development of goblet cells in the broiler jejunum. A total of 480 1-day-old Arbor Acres broilers were randomly allotted to 5 treatments with 6 replications of 16 chicks each. Birds were fed with low selenium corn-soybean meal-based diets supplemented with 0.1, 0.2, 0.3, or 0.4 mg/kg of SeNPs. On d 21, dietary supplementation of SeNPs effectively reduced the mortality of broilers. The villus height and the villus height/crypt depth ratio of the jejunum showed significant quadratic effects with the increasing concentration of SeNPs (*P* < 0.05). The mRNA expression of zonula occluden-1 (*ZO-1*), *ZO-2, claudin-3*, and *claudin-5* in the jejunum decreased linearly with the increasing dose of SeNPs (*P* < 0.05). The mRNA expression levels of interleukin 1 beta (*IL-1*β), *IL-18*, and the concentration of reactive oxygen species (ROS) in the jejunum decreased linearly with the increase of SeNPs concentration (*P* < 0.05). Compared with the control group, the number of goblet cells in the jejunum was significantly increased by adding 0.1 and 0.4 mg/kg SeNPs(*P* < 0.05). In addition, the mRNA expression of Mucin2 (*Muc2*) showed a significant quadratic relationship that increased after adding 0.1 mg/kg SeNPs (*P* < 0.05). Dietary SeNPs also linearly reduced the expression of v-myc avian myelocytomatosis viral oncogene homolog (*c-myc*) (*P* < 0.05). The mean density of TUNEL positive cells in the 0.2 and 0.4 mg/kg SeNPs groups were lower than the control group (*P* < 0.05). Similarly, the mRNA expression levels of B-cell lymphoma-2 (*Bcl-2*), Bcl-2-associated X (*Bax*), NLR family pyrin domain containing 3 (*NLRP3*), cysteinyl aspartate specific proteinase-1 (*Caspase-1*), toll-like receptor-2 (*TLR-2*), and myeloid differentiation factor 88 (*MyD88*) in the jejunum decreased linearly with the increase of SeNPs concentration (*P* < 0.05). Results show that supplementation with 0.2 mg/kg SeNPs may decrease intestinal oxidative stress and inflammation by modifying the activation of NLRP3 signaling pathway, which can effectively promote intestinal goblet cells of 21-day-old broilers.

## Introduction

Selenium (Se), as an essential micronutrient, plays an important role in the health of humans and animals. Selenium is a well-known antioxidant and exerts biological function by synthesizing selenium-containing proteins (selenoproteins), which contain at least one selenocysteine (Sec) (1). Moreover, Sec is the active site of selenoproteins such as glutathione peroxidase (GSH-Px) and thioredoxin reductase (TrxR), which can make them suitable for oxidation/reduction reactions and prevent cellular oxidative damage (2, 3). The chemical forms of Se in nature are either inorganic (Se, Na_2_SeO_3_, Na_2_SeO_4_) or organic (selenomethionine/selenocysteine) (4, 5). Compared with selenomethionine and sodium selenite, selenium nanoparticles (SeNPs) transformed from sodium selenite have stronger antioxidant activities and lower cytotoxicity, thereby effectively protecting human and animal intestinal epithelial cells (6). Due to its high bioavailability, anticancer, and antimicrobial properties; SeNPs are widely used in nanomedicine and agriculture (7, 8). Therefore, SeNPs are regarded as a potential and novel Se nutritional supplement (8).

Chicks are exposed to a large number of pathogenic bacteria after hatch (9), and the intestine is more susceptible to oxidative stress, which can increase the level of intestinal inflammation. Intestinal health of broilers is largely achieved by function of the intestinal barrier. The intestinal mucus layer provides the first line of defense for the intestine. The inner mucus layer prevents the invasion of pathogenic bacteria, and the outer mucus layer provides a place and nutrients for symbiotic bacteria (10). The main component of the intestinal mucus layer is the Mucin2 (Muc2), which is primarily secreted by goblet cells (10). Recent research has indicated that the secretion of mucin from intestinal goblet cells depends on several intersecting cellular processes. These include endocytosis, autophagy, reactive oxygen species (ROS) generation, and inflammasome assembly and activation (11). When pathogenic bacteria invade the intestine, goblet cells non-specifically endocytose Toll-like receptor (TLR) ligands by triggering the NLR family pyrin domain containing 6 (NLRP6) inflammasome downstream of TLR- and MyD88-dependent ROS synthesis, which induces Muc2 exocytosis (12). Meanwhile, the level of inflammatory factors such as interleukin 9 (IL-9), IL-13, and IL-25 also increase, which promote the differentiation and proliferation of goblet cells through the STAT6 signaling pathway (13). Therefore, the development of goblet cells can effectively promote the formation of the mucus layer to protect the intestinal tissues.

Selenium promotes the proliferation of goblet cells by the NF-E2-related factor 2 (Nrf2) signaling pathway and ameliorates the structure of intestinal mucus protein, protecting the intestinal barrier function against oxidative damage (14). Selenium deficiency induces intestinal cell apoptosis and causes serious damage to the morphology and function of the intestinal mucosa (15, 16). SeNPs, an effective antioxidant, can decrease the level of intestinal inflammatory factors and reduce the production of intestinal ROS to improve the intestinal barrier function (17, 18). However, it is not clear whether SeNPs can promote the development of intestinal goblet cells in chicks to enhance intestinal barrier functions.

In this study, we investigated the histological characteristics, antioxidant activities, goblet cell differentiation factors, inflammatory factors, and pyroptosis signaling pathways in the jejunum of chicks fed with increasing doses of SeNPs, as to elucidate the molecular mechanism of the possible SeNPs-induced intestinal goblet cells development.

## Materials and Methods

All procedures and protocols were approved by the Animal Ethics Committee of the China Agricultural University (Permit Number: AW02211202-1-2).

### Animals and Experimental Design

480 1-day-old Arbor Acres broilers were randomly divided into 5 dietary treatments (each involving 6 replicate cages with 16 birds each). Low-Se corn (the Se deficient region of Heilongjiang Province, China) was used in the diet, and the basal diet without Se in the trace elements was used as the blank control group. The basal diet was formulated to meet or exceed the nutrient requirements for broilers recommended by National Research Council (1994). The ingredient and nutrient compositions of the basal diets are shown in Table 1. The experimental group was supplemented with 0.10, 0.20, 0.30 and 0.40 mg/kg SeNPs. The analyzed selenium content of the 5 experimental diets was 0.10, 0.19, 0.25, 0.37, and 0.43 mg/kg for the starter phase (0 to 21 d). The experimental period was 21 days. All diets were pelleted and crumbled.

**Table 1:** Ingredients and composition of the basal experimental diets.

**Ingredients, %**	**Starter diet**
Corn	50.40
Soybean meal	35.40
Corn gluten meal	5.00
Corn oil	2.80
Wheat flour	2.00
Dicalcium phosphate	1.75
Limestone	1.20
Salt	0.35
Trace mineral premix^a^(No sodium selenite)	0.20
Vitamin premix^b^	0.03
Choline chloride (50%)	0.20
DL-Methionine	0.26
L-Lysine HCL	0.26
Antioxidant	0.02
Phytase	0.03
Nutrient composition, %^c^	
ME (kcal/kg)	2998
CP, %	22.89
Lysine, %	1.30
Methionine, %	0.58
Methionine+Cysteine, %	0.93
Threonine,%	0.85
Tryptophan,%	0.26
Calcium, %	0.95
NPP, %	0.40

a *The trace mineral premix provided the following per kg of diets: Cu,16 mg (as CuSO_4_·5H_2_O); Zn, 110 mg (as ZnSO_4_); Fe, 80 mg (as FeSO_4_·H_2_O); Mn, 120 mg (as MnSO_4_·H_2_O); I, 1.5 mg (as CaIO_3_); Co, 0.5 mg (CoCl_2_·H_2_O)*.

b *The vitamin premix provided the following per kg of diets: vitamin A, 10 000 IU; vitamin D_3_, 3600 IU; vitamin E, 20 mg;vitamin K_3_, 2 mg; vitamin B_1_, 2 mg; vitamin B_2_, 6.4 mg; VB_6_, 3 mg;VB_12_, 0.02 mg; biotin, 0.1 mg; folic acid, 1 mg; pantothenic acid,10 mg; nicotinamide, 30 mg*.

c *All the nutrient levels are calculated values*.

The SeNPs were provided by the Beijing Wahmix Bio-technology Co. Ltd. (Tangshan, China). SeNPs were transformed from sodium selenite by the unmodified *Bacillus subtilis* S12 *via* fermentation, which converted sodium selenite into red SeNPs with a diameter of 170 nm. The proportion of SeNPs was 98%, and the SeNPs premix was created by mixing the raw product with starch for a final Se content of 3,694 mg/kg.

### Sample Collection

Incidence of mortality was recorded daily during the experimental period. On d 21, 6 birds (one bird per cage) per treatment were randomly selected, electrically stunned, and euthanized by cervical dislocation. Samples of the middle jejunum (1 cm) were excised for morphological analysis. Three pieces of the middle jejunum (0.5 cm) samples were collected and cleaned with sterile PBS, rapidly frozen in liquid nitrogen, and transferred to −80 °C for RNA and protein analysis.

### The histological characteristics of Jejunum Tissues

The formalin-fixed paraffin-embedded jejunum tissues were sectioned (5–6 μm) using a microtome and adhered to glass slides. Samples were deparaffinized with xylene and washed in 100% ethanol. Cells expressing mucin glycoprotein were identified using a combined periodic acid Schiff stain (**PAS**) staining. Under the Leica microscope (DMI803250593, Heidelberg, Germany), the intestinal villus height (VH) and crypt depth (CD) were measured by randomly selecting 10 intact villi for every bird. The density of goblet cells was expressed as the number of goblet cells per 100 μm (19). The method was calculated by randomly selecting 10 intact villi and measuring the number of goblet cells in corresponding villi under the mid-villi area (100 μm in length) (19).

### Validation of Differentially Expressed Gene by Quantitative RT-PCR

The total RNA was isolated from the jejunum by TRIzol reagent according to the manufacturer's protocol (9109, TaKaRa, Tokyo, Japan). RNA quantity was determined using a NanoDrop 2000 spectrophotometer (Thermo Fisher, Waltham, MA, USA). Then, first-strand cDNA was synthesized using PrimeScript™ RT reagent Kit (RR047A, TaKaRa). One-step real-time PCR was performed with the 2 × RealStar Green Fast Mixture (A301-05, GenStar, Beijing, China) using an CFX96 Touch fluorescence quantitative PCR instrument (Bio-Rad, California, USA) in accordance with the manufacturer's guidelines. The primer pairs were designed based on genomic data of *Gallus domesticus* in NCBI. Primer sequences of the *GAPDH, claudin-1, claudin-3, claudin-5*, zonula occluden-1 (***ZO-1***), ***ZO-2***; Mucin2 (*Muc2*); B-cell lymphoma-2 (***Bcl-2***), cysteinyl aspartate specific proteinase-3 (*Caspase-3*), and Bcl2-associated X (*Bax*) used in the study were listed in Table 2. All the measurements were carried out in triplicate (*N* = 6, the cage was used as experimental unit) and the average values were calculated. The relative transcription levels of genes in different groups were calculated relative to *GAPDH* (the normalizer) using the 2 ^−ΔΔCT^ method.

**Table 2:** Sequence of the oligonucleotide primers used forquantitative real-time PCR.

Gene^*^	**Primer sequence (5' → 3')**	**Genebank accession**
*ZO-1*	F:CTTCAGGTGTTTCTCTTCCTCCTC	XM_413773
	R:CTGTGGTTTCATGGCTGGATC	
*ZO-2*	F:TGTCTGCGTGGTTGTTCCAT	XM_025144668.2
	R:CACTCACAAGGAGACGGCAG	
*Claudin−1*	F:CTGATTGCTTCCAACCAG	NM_001013611
	R:CAGGTCAAACAGAGGTACAAG	
*Claudin−3*	F:GGACACCATGTCTATGGGGC	NM_204202.1
	R:TCACGATGTTGTTGCCGATG	
*Claudin−5*	F:GAGATCTTTGTGCCCTGGCT	NM_204201.2
	R:TAGCCTAAGCATCACGAGCG	
*Mucin2*	F:TCACCCTGCATGGATACTTGCTCA	NM_001318434.1
	R:TGTCCATCTGCCTGAATCACAGGT	
*Klf4*	F:TCAAGGCACACCTGAGAACC	XM_004949369.4
	R:GCCCGTGTGTTTTCGGTAAT	
*Nrf2*	F:ACGCTTTCTTCAGGGGTAGC	NM_205117.1
	R:GGCAAGGCAGATCTCTTCCAA	
*c-myc*	F:ACACAACTACGCTGCTCCTC	NM_001030952.1
	R:CTCCTCTGAGTCTGACGTGC	
*Bax*	F:ATCGTCGCCTTCTTCGAGTT	XM_204725
	R:ATCCCATCCTCCGTTGTCCT	
*Bcl-2*	F:TCGCGCCGCTACCAGAGGGACTTC	NM_205339
	R:CCGGTTGACGCTCTCGACGCACAT	
*Caspase-3*	F:GGCTCCTGGTTTATTCAGTCTC	NM_204725.1
	R:ATTCTGCCACTCTGCGATTT	
*IL-1β*	F:TCTGCCTGCAGAAGAAGCC	NM_204524.1
	R:CTCCGCAGCAGTTTGGTCAT	
*IL-6*	F:CAAGAAGTTCACCGTGTGCG	NM_204628.1
	R:GGAGAGCTTCGTCAGGCATT	
*IL-8*	F:GCCAAGGCTCAGCTCAATTC	NM_205498.1
	R:GCCAAGGCTCAGCTCAATTC	
*NLRP3*	F:TGGTGTGAGGATGCTCTGTG	NM_001348947.1
	R:GACAGGTCCAGCTCCTCCA	
*Caspase-1*	F:CTGCCGTGGAGACAACATAG	XM_015295935.1
	R:AGGAGACAGTATCAGGCGTGGAAG	
*IL-18*	F:TGATGAGCTGGAATGCGATG	NM_204608.2
	R:ACTGCCAGATTTCACCTCCTG	
*TLR2*	F:GATTGTGGACAACATCATTGACTC	NM_001161650
	R:AGAGCTGCTTTCAAGTTTTCCC	
*TLR4*	F:CCACTATTCGGTTGGTGGAC	NM_001030693.1
	R:ACAGCTTCTCAGCAGGCAAT	
*MyD88*	F:CCGTATGGGCATGGAACAGA	NM_001030962.4
	R:CTGGCAAGACATCCCGATCA	
*GADPH*	F:ATGGCATCCAAGGAGTGAGC	NM_204305.1
	R:GGGAACAGAACTGGCCTCTC	

*: *F, forward primer; R, reverse primer; primers were synthesized by Biotech (Shanghai) Co., Ltd*.

### TUNEL Assay

Jejunum tissue specimens were embedded in paraffin and sectioned at 5 μm for processing by the TUNEL method using the DAB (SA-HRP) Tunel Cell Apoptosis Detection Kit (G1507, Servicebio Technology, Wuhan, China) in accordance with the manufacturer's guidelines. A total of 5 representative randomly chosen, non-adjacent, non-overlapping fields were counted for each TUNEL-stained tissue sample. Specimens were evaluated by the Leica microscope at 400× magnification for cell counting. ImageJ software (National Institute of Health) was used to assess the area and density of dyed region, and the integrated option density (IOD) value of the TUNEL-stained section. Finally, we calculated the mean density (20).

### Measurements of the Goblet Cell Differentiation Factors, Inflammatory Factors and Pyroptosis Genes by q-PCR

The mRNA levels of Krüppel-like factor 4 (*Klf4*), NF-E2-related factor 2 (*Nrf2*), v-myc avian myelocytomatosis viral oncogene homolog(*c-myc*); interleukin 1 beta (*IL-1*β), *IL-6, IL-8, IL-18*; NLR family pyrin domain containing 3 (*NLRP3*), cysteinyl aspartate specific proteinase-1 (*Caspase-1*), toll-like receptor-2 (*TLR-2*), *TLR-4*, and myeloid differentiation factor88 (*MyD88*) genes were evaluated by quantitative RT-PCR. Primer sequences used in the study were listed in Table 2. All the measurements were carried out in triplicate (*N* = 6, the cage was used as experimental unit) and the average values were calculated. Expression levels of the target gene were evaluated using a relative quantification approach (2^−Δ*ΔCT*^ method) against *GAPDH* levels.

### Measurements of the Goblet Cell Differentiation Factors, Inflammatory Factors and Pyroptosis Protein by Western Blotting

Western blotting for differentially expressed proteins was performed according to a previously reported method (21). Jejunum tissue extracts were prepared with the RIPA buffer. Proteins were separated by 10% SDS-PAGE gel; separated proteins were transferred onto PVDF membranes (IPVH00010, Millipore, Massachusetts, USA) and were probed with the following primary antibodies: Anti-c-Myc Mouse mAb (PTM-5028, Jingjie PTM BioLab, Hangzhou, China); Caspase 1 Antibody (AF5418, Affinity Biosciences, Liyang, China); and Anti-beta Actin Rabbit mAb (PTM-5143, Jingjie PTM BioLab). At last, the blots were visualized by the BeyoECL Plus (P0018S, Beyotime, Shanghai, China). The representative blot from four independent experiments was presented for each protein. The relative levels of protein expression were calculated using densitometric scans by ImageJ software and were normalized to the GAPDH levels from four independent experiments. For each independent experiment, the relative levels of protein expression were calculated.

### Measurements of Reactive Oxygen Species in Jejunum Tissue by ELISA

100 mg jejunum tissue was weighed and homogenized with 900 μL PBS, and the supernatant was collected after centrifugation at 845 g for 15 min. The supernatant was measured using chicken reactive oxygen species (ROS) ELISA Kit (MM-6012001, Jiangsu MEIMIAN, Yancheng, China) based on the manufacturer's instructions. Then, the concentration was normalized by the weight of the jejunum sample and expressed as pg/mL of tissue.

### Statistical Analysis

Data were statistically analyzed by one-way analysis of variance (ANOVA) using SPSS 20.0 software (SPSS Inc. Chicago, IL). All data were tested for homogeneity of variances using Levene's test. We analyzed different supplemented concentrations of SeNPs to the basal diet to evaulate linear or quadratic responses. The significance among the groups was identified using the Duncan test for multiple comparisons. Results were presented as means ± SEM. Significance was accepted at *P* < 0.05. The regression equation was calculated by Microsoft Excel (2016).

## Results

### Effects of SeNPs on the Mortality of Broilers

**Figure 1:**
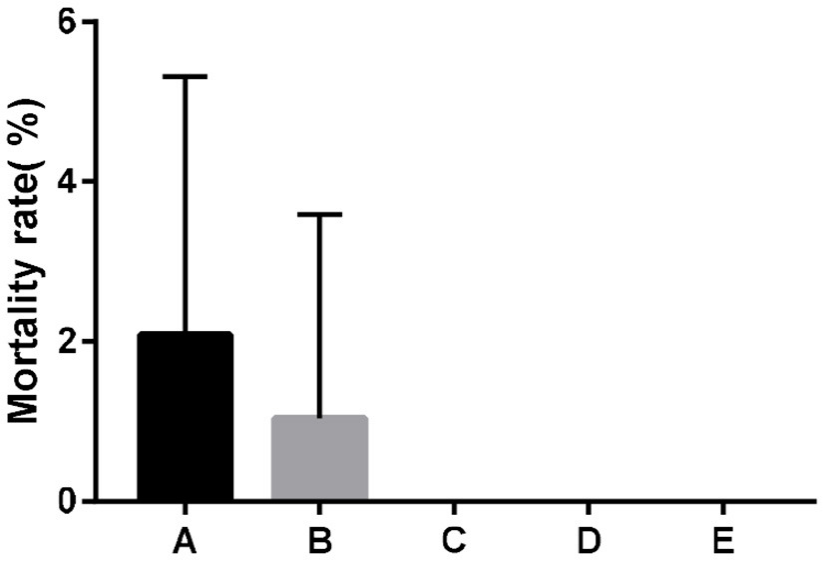
Effects of SeNPs on the mortality of broilers. A, B, C, D, E represent the basal diet supplemental with 0, 0.1, 0.2, 0.3, 0.4 mg/kg SeNPs, respectively. All data were presented as mean ± SEM (*n* = 6).

Dietary supplementation of SeNPs could effectively reduce the mortality of broilers (Figure 1).

### Effects of SeNPs on Jejunal Morphology

**Figure 2:**
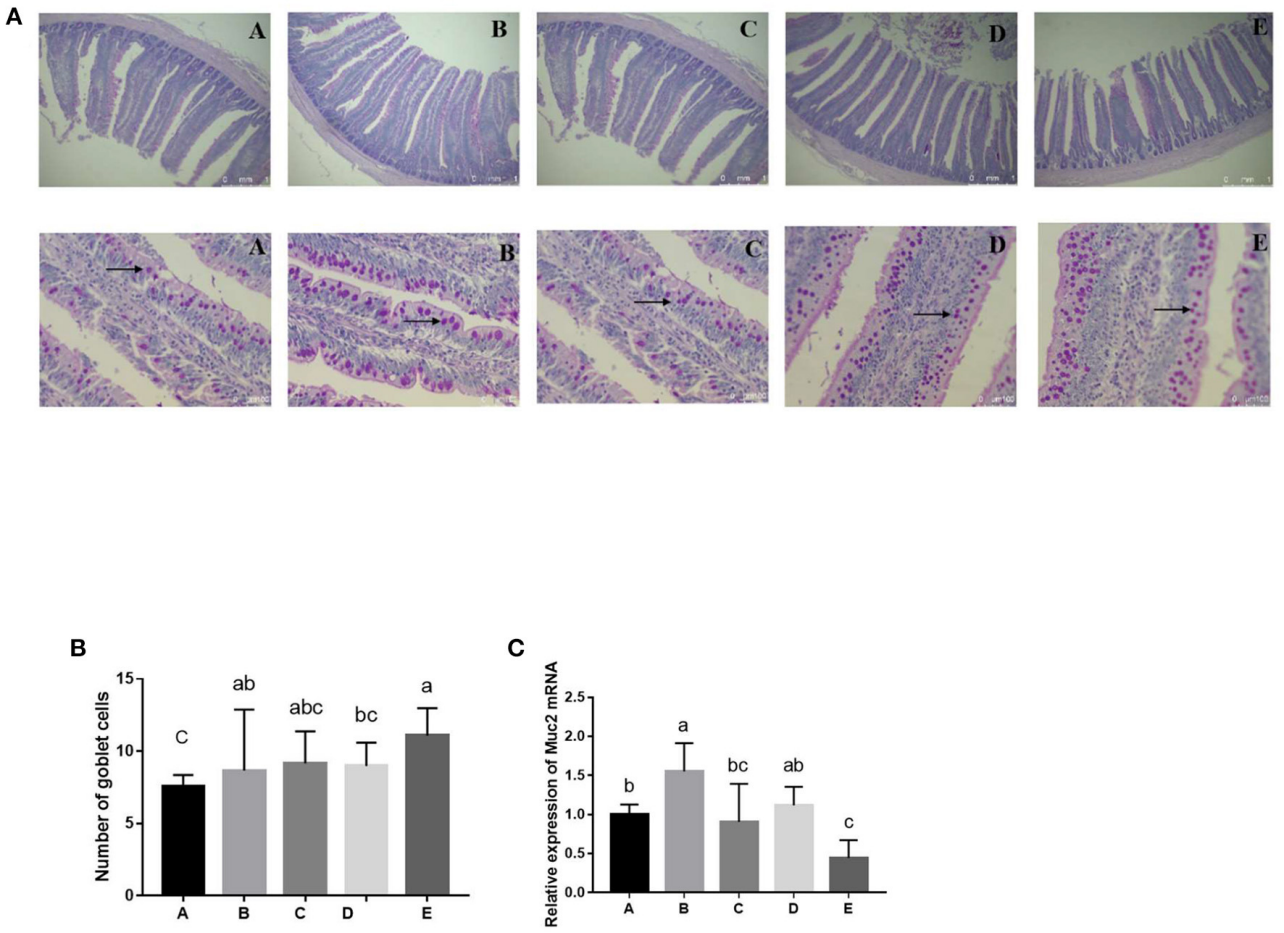
Effects of SeNPs on goblet cells of jejunum. **(A)** The staining of goblet cells (black arrow) by the PAS method. **(B)** The number of goblet cells in different groups. **(C)** mRNA levels of *Muc2* in jejunum analyzed by real-time qPCR. A, B, C, D, E represent the basal diet supplemental with 0, 0.1,0.2,0.3,0.4 mg/kg SeNPs, respectively. All data were presented as mean ± SEM (n = 6). ^a,b,c^ means without common superscripts differ significantly (*P* < 0.05). SeNPs, selenium nanoparticles; PAS, periodic acid Schiff stain; Muc2, mucin 2.

The VH (y = −7824.9x^2^ + 3412.9x + 1529.6, R^2^ = 0.8042, *P* < 0.05) and the VH/CD ratio (y = −36.357x^2^ + 19.273x + 7.1029, R^2^ = 0.8222, *P* < 0.05) of the jejunum showed a significant quadratic effect with increasing the concentration of SeNPs (Table 3, Figure 2A). The VH of jejunum reached the highest value after adding 0.1 mg/kg SeNPs, and the VH/CD of jejunum reached the highest value after adding 0.30 mg/kg SeNPs.

**Table 3:** Effects of SeNPs on jejunal morphology of broilers.

**Items**	**SeNPs(mg/kg)**					**Statistic**			
	**0**	**0.1**	**0.2**	**0.3**	**0.4**	**SEM**	***P*** **-value**	**Linear**	**Quadratic**
VH (μm)	1,479.60^b^	1,912.00^a^	1,841.60^a^	1,806.83^a^	1,673.67^ab^	50.005	0.049	0.516	0.008
CD (μm)	204.96	229.97	201.14	178.13	200.30	7.445	0.269	0.218	0.955
VH/CD	7.31^c^	8.36^bc^	9.18^ab^	10.35^a^	8.68^abc^	0.301	0.016	0.019	0.022

a, b, c *Means in the same row without common superscripts differ significantly (P < 0.05)*.

### Effects of SeNPs on mRNA Expression of Jejunal Barrier Protein-Related Genes

The mRNA expression of *ZO-1* (y = −1.7818x + 0.8816, R^2^ = 0.855, *P* < 0.01), *ZO-2* (y = −1.5657x + 0.8018, R^2^ = 0.6574, *P* < 0.05), *claudin-3* (y = −1.8927x + 0.945, R^2^ = 0.9657, *P* < 0.01), and *claudin-5* (y = −1.9663x + 0.7722, R^2^ = 0.7277, *P* < 0.01) in the jejunum decreased linearly with the increasing dose of SeNPs (Table 4). Compared with the control group, the mRNA expressions of *claudin-1* in the jejunum were increased by adding 0.1 mg/kg SeNPs (*P* < 0.01), and the mRNA expression levels were significantly reduced by adding 0.2 and 0.3 mg/kg SeNPs.

**Table 4:** Effects of SeNPs on the mRNA expression of jejunal barrier protein-related genes of broilers.

**Items**	**SeNPs(mg/kg)**					**Statistic**			
	**0**	**0.1**	**0.2**	**0.3**	**0.4**	**SEM**	***P*** **-value**	**Linear**	**Quadratic**
*ZO-1*	1.00^a^	0.59^b^	0.41^bc^	0.46^b^	0.17^c^	0.064	<0.001	<0.001	0.124
*ZO-2*	1.00^a^	0.35^b^	0.52^b^	0.35^b^	0.22^b^	0.083	0.016	0.004	0.254
*claudin-1*	1.00^b^	1.24^a^	0.57^c^	0.55^c^	0.99^b^	0.063	<0.001	0.024	0.001
*claudin-3*	1.00^a^	0.66^b^	0.58^b^	0.41^bc^	0.18^c^	0.065	0.001	<0.001	0.790
*claudin-5*	1.00^a^	0.37^b^	0.36^b^	0.36^b^	0.19^b^	0.071	<0.001	<0.001	0.024

a, b, c *Means in the same row without common superscripts differ significantly (P < 0.05)*.

### Effects of SeNPs on the Numbers of Goblet Cells and the mRNA Level of Muc2 of the Jejunum

Compared with the control group, the number of goblet cells in the jejunum was significantly increased by adding 0.1 and 0.4 mg/kg SeNPs (*P* < 0.01) (Figures 2A,B). In addition, the mRNA expression of *Muc2* (y = −8.9205x^2^ + 2.3609x + 0.974, R^2^ = 0.7224, *P* < 0.01) showed a significant quadratic relationship that increased after adding 0.1 mg/kg SeNPs (Figure 2C).

### Effects of SeNPs on Goblet Cell Differentiation Regulator of Jejunal

**Figure 3:**
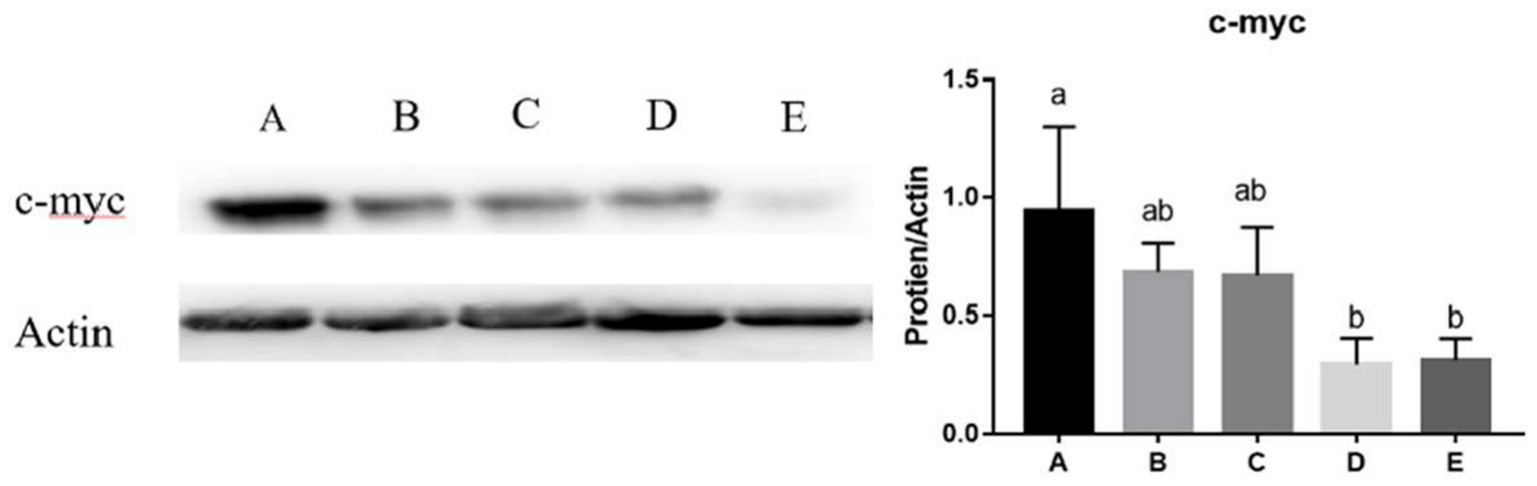
Effects of SeNPs on the protein expression of jejunal c-myc. A, B, C, D, E represent the basal diet supplemental with 0, 0.1, 0.2, 0.3, 0.4 mg/kg SeNPs, respectively. Values are presented as mean ± SEM (*n* = 4). ^a,b^ Significant differences are indicated by different letters (*P* < 0.05). SeNPs, selenium nanoparticles.

The mRNA expression of *Klf4* (y = −1.64x + 1.072, R^2^ = 0.9264, *P* < 0.05) and *c-myc* (y = −1.4836x + 1.0333, R^2^ = 0.6843, *P* < 0.01) in the jejunum decreased linearly with the increasing dose of SeNPs (Table 5). Compared with the control group, the mRNA expression of *Nrf2* in the jejunum was significantly decreased by adding 0.2 and 0.4 mg/kg SeNPs (*P* < 0.01) (Table 5). Moreover, dietary SeNPs also decreased linearly the protein expression level of c-myc (y = −1.6389x + 0.9075, R^2^ = 0.8976, *P* < 0.01) (Figure 3).

**Table 5:** Effects of SeNPs on goblet cell differentiation regulator of jejunum.

**Items**	**SeNPs(mg/kg)**					**Statistic**			
	**0**	**0.1**	**0.2**	**0.3**	**0.4**	**SEM**	***P*** **-value**	**Linear**	**Quadratic**
*Klf4*	1.00^a^	0.95^ab^	0.85^ab^	0.53^bc^	0.39^c^	0.076	0.029	0.002	0.498
*Nrf2*	1.00^a^	1.00^a^	0.50^b^	0.99^a^	0.47^b^	0.072	0.005	0.013	0.986
*c-myc*	1.00^a^	0.97^ab^	0.57^bc^	0.82^ab^	0.33^c^	0.071	0.004	0.001	0.579

a, b, c *Means in the same row without common superscripts differ significantly (P < 0.05)*.

### Effects of SeNPs on the Inflammation and Oxidative Stress of the Jejunum

The mRNA expression of *IL-1*β (y = −1.9663x + 0.7722, R^2^ = 0.7277, *P* < 0.01) and the concentration of ROS (y = −1.5657x + 0.8018, R^2^ = 0.6574, *P* < 0.01) in the jejunum decreased linearly with the increase of SeNPs concentration (Table 6). Compared with the control group, the mRNA expressions of *IL-8* in the jejunum were significantly increased by adding 0.1 mg/kg SeNPs (*P* < 0.05) (Table 6).

**Table 6:** Effects of SeNPs on the inflammation and oxidative stress of the jejunum.

**Items**	**SeNPs(mg/kg)**					**Statistic**			
	**0**	**0.1**	**0.2**	**0.3**	**0.4**	**SEM**	***P*** **-value**	**Linear**	**Quadratic**
*IL-1β*	1.00^a^	0.41^b^	0.19^c^	0.14^c^	0.15^c^	0.068	<0.001	<0.001	<0.001
*IL-6*	1.00^a^	0.65^ab^	0.25^b^	0.39^b^	0.54^ab^	0.091	0.099	0.05	0.054
*IL-8*	1.00^b^	1.57^a^	0.98^b^	0.83^b^	1.34^ab^	0.089	0.021	0.611	0.534
ROS (pg /mL)	171.87^b^	199.89^a^	119.90^c^	125.04^c^	106.20^c^	7.079	<0.001	<0.001	0.869

a, b, c *Means in the same row without common superscripts differ significantly (P < 0.05)*.

### Effects of SeNPs on Apoptosis of the Jejunum

**Figure 4:**
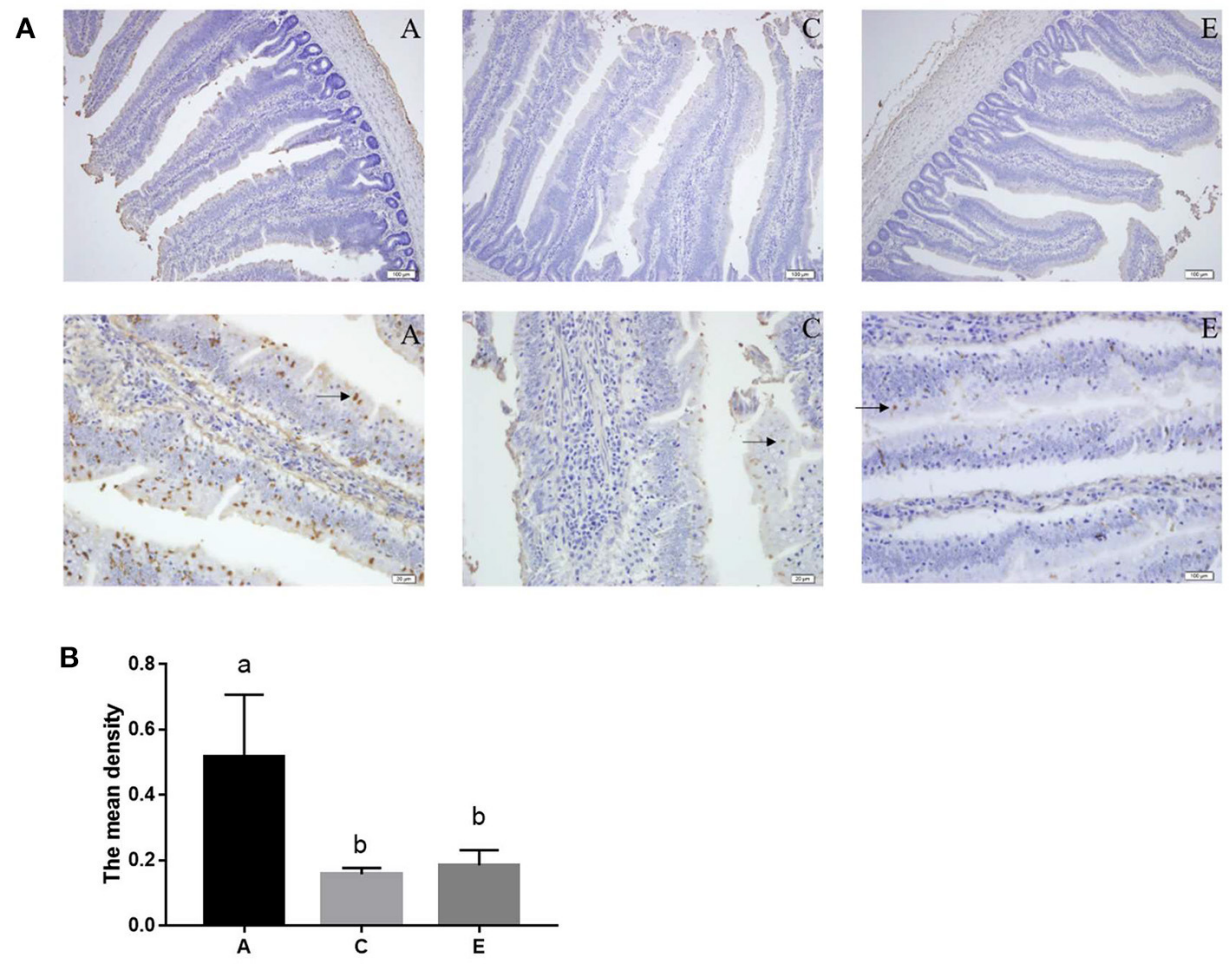
Effects of SeNPs on apoptosis of jejunum. **(A)** The staining of apoptotic cells (black arrow) by the TUNEL method. **(B)** The mean density of the TUNEL-stained section in different groups. A, C, E represent the basal diet supplemental with 0, 0.2, 0.4 mg/kg SeNPs, respectively. Values are presented as mean ± SEM (*n* = 6). ^a,b^ Significant differences are indicated by different letters (*P* < 0.05). SeNPs, selenium nanoparticles.

As shown in Figure 4, the mean density of TUNEL positive cells in the 0.2 and 0.4 mg/kg SeNPs group were significantly lower than the control group. Furthermore, by detecting apoptosis-related genes, we also found that the mRNA expression of *Bax* (y = −1.3905x + 0.828, R^2^ = 0.6831, *P* < 0.01) and *Bcl-2* (y = −2.2442x + 0.8208, R^2^ = 0.8149, *P* < 0.01) in the jejunum decreased linearly with the increasing dose of SeNPs (Table 7).

**Table 7:** Effects of SeNPs on mRNA expression of apoptosis genes of the jejunum.

**Items**	**SeNPs(mg/kg)**					**Statistic**			
	**0**	**0.1**	**0.2**	**0.3**	**0.4**	**SEM**	***P*** **-value**	**Linear**	**Quadratic**
*Bax*	1.00^a^	0.49^b^	0.45^b^	0.51^b^	0.29^b^	0.063	0.001	<0.001	0.108
*Caspase-3*	1.00	0.85	0.82	0.96	0.75	0.039	0.224	0.145	0.982
*Bcl-2*	1.00^a^	0.38^bc^	0.40^b^	0.02^d^	0.06^cd^	0.082	<0.001	<0.001	0.040

a, b, c, d *Means in the same row without common superscripts differ significantly (P < 0.05)*.

### Effects of SeNPs on Pyroptosis-Related Genes

**Figure 5:**
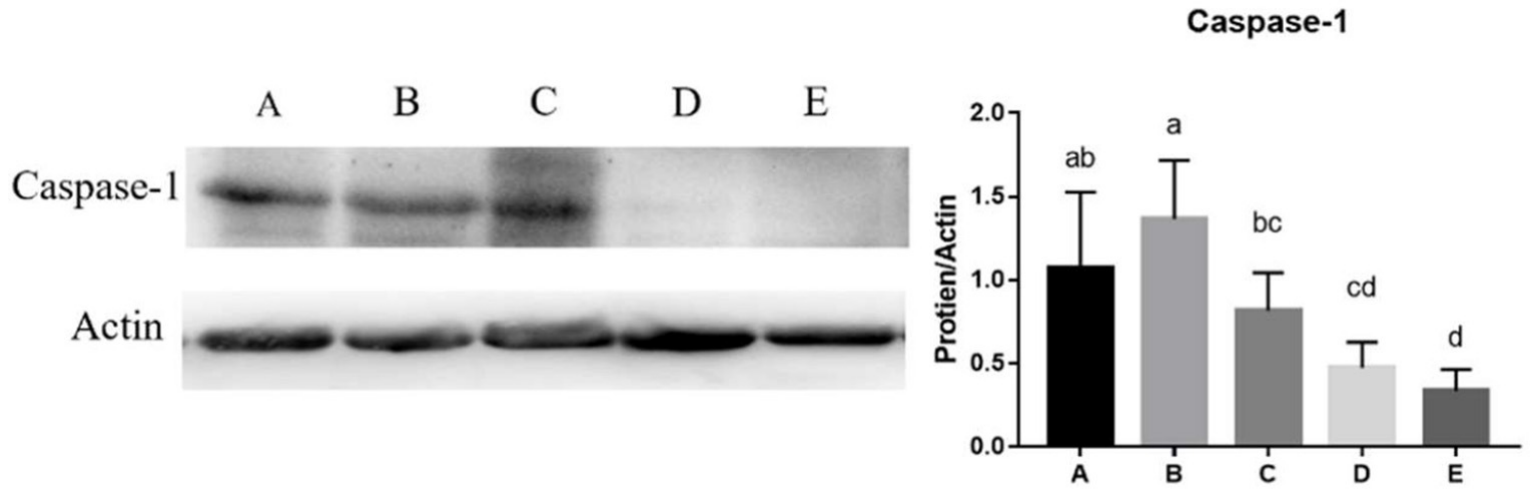
Effects of SeNPs on the protein expression of jejunal Casepase-1. A, B, C, D, E represent the basal diet supplemental with 0, 0.1, 0.2, 0.3, 0.4 mg/kg SeNPs, respectively. Values are presented as mean ± SEM (*n* = 6). ^a,b,c,d^ Significant differences are indicated by different letters (*P* < 0.05). SeNPs, selenium nanoparticles.

The mRNA expression levels of *NLRP3* (y = −1.2898x + 0.8725, R^2^ = 0.761, *P* < 0.05), *Casepase-1*(y = −1.2214x + 1.0971, R^2^ = 0.671, *P* < 0.05), *IL-18*(y = −1.2262x + 0.8488, R^2^ = 0.6945, *P* < 0.05), *TLR2*(y = −1.6146x + 0.8663, R^2^ = 0.8449, *P* < 0.05), and *MyD88* (y = −1.8012x + 0.9246, R^2^ = 0.911, *P* < 0.05) in the jejunum decreased linearly with the increase of SeNPs concentration (Table 8). Compared with the control group, the mRNA expressions of *TLR4* in the jejunum were significantly decreased by adding 0.1, 0.2 and 0.4 mg/kg SeNPs (*P* < 0.05) (Table 8). Moreover, as shown in Figure 5, we found that dietary SeNPs decreased linearly the protein expression level of Casepase-1 (y = −0.2362x + 1.5208,R^2^ = 0.7799, *P* < 0.01).

**Table 8:** Effects of SeNPs on Pyroptosis-related genes.

**Items**	**SeNPs(mg/kg)**					**Statistic**			
	**0**	**0.1**	**0.2**	**0.3**	**0.4**	**SEM**	***P*** **-value**	**Linear**	**Quadratic**
*NLRP3*	1.00^a^	0.63^b^	0.48^b^	0.56^b^	0.39^b^	0.062	0.004	<0.001	0.145
*Casepase-1*	1.00^ab^	1.19^a^	0.72^bc^	0.76^bc^	0.60^c^	0.065	0.006	0.002	0.454
*IL-18*	1.00^a^	0.62^b^	0.45^b^	0.49^b^	0.45^b^	0.060	0.019	0.005	0.050
*TLR2*	1.00^a^	0.54^b^	0.52^b^	0.39^b^	0.27^b^	0.072	0.003	<0.001	0.187
*TLR4*	1.00^a^	0.54^b^	1.17^a^	0.54^b^	0.42^b^	0.092	0.008	0.018	0.192
*Myd88*	1.00^a^	0.74^a^	0.42^b^	0.38^b^	0.28^b^	0.069	<0.001	<0.001	0.141

a, b, c *Means in the same row without common superscripts differ significantly (P < 0.05)*.

## Discussion

Selenium supplementation is necessary for optimal growth of broilers (22, 23). It is believed that supplementing higher levels of Se could provide reduced mortality associated with marginal selenium deficiency syndrome (24). Under the conditions of this study, the results indicated that dietary SeNPs could reduce the mortality of broilers. Similarly, Zhou and Wang (25) also found that dietary SeNPs could significantly reduce broiler mortality. In commercial production, chicks may encounter challenges of pathogenic bacteria and oxidative stress, and the addition of SeNPs in the diet may reduce levels of inflammation and improve antioxidant status to improve the growth of broiler.

### Intestinal Morphology and Intestinal Tight Junction

Changes in the intestinal morphology are related to the development of intestine and the surface area for nutrient absorption (26, 27). In the current trial, the VH of jejunum reached the highest value after adding 0.1 mg/kg SeNPs, and the VH/CD of jejunum reached the highest value after adding 0.3 mg/kg SeNPs. These were in agreement with the previous findings of Ali et al. (28) and Khajeh Bami et al. (29). The authors found that dietary SeNPs supplementation produced positive effects on some VH and VH/CD in the intestine of broilers. Dietary Se supplementation can promote intestinal structure by regulating intestinal flora (30). Tang et al. (31) found that the modified intestinal flora provided nutrition and stimulated the growth of intestinal villi. On the other hand, Se regulates the activity of inflammatory cytokines and increases the antioxidant status to promote the development of intestinal tissues (32). Subsequent experimental results also showed that SeNPs could effectively reduce levels of oxidative stress and inflammation in the intestine. Thus, we speculate that SeNPs can reduce the growth of pathogenic intestinal microbes and reduce the level of intestinal inflammation to improve the intestinal morphology.

Tight junctions (TJs) not only connect cells but also form channels that allow penetration between cells, resulting in varying epithelial surface tightness (33). Expression of Claudin-1,−3, and−5 leads to a very tight epithelia, coinciding with an increased transepithelial electrical resistance (TER) and decreased solute permeability (mainly sodium ions) across the epithelial monolayer (34, 35). The upregulation of tight junction proteins increased intestinal integrity and limited paracellular permeability, thus essentially helping the epithelial barrier to prevent pathogens from entering the body (36). However, TJs represent receptors for the toxins of pathogen (*Clostridium perfringens* enterotoxin) (36). Pathogenic bacteria utilized a subset of TJs as receptors to form pores in the membrane of intestinal epithelial cells, whereby Ca influx through these pores ultimately triggers intestinal cell injury (36). Therefore, the up- or down-regulation of tight junction proteins is difficult to interpret, which also the need to assess other factors (the microbial community) that affect gut health. The results of the present study showed that supplementation of SeNPs reduced the ZO-1, ZO-2, claudin-3, and claudin-5 relative expression in the jejunum compared to control diet at 21 days of age. Similarly, the expression of intestinal claudin-1, ZO−1, and ZO-2 was significantly reduced in diets supplemented with SeNPs (28). Ali et al. (28) found that the reduction of claudin-1, ZO-1, and ZO-2 by SeNPs might reduce the sites to attaching the pathogen bacteria. Meanwhile, the products of oxidative stress also increased intestinal permeability by decreasing expression levels of tight junction proteins (19, 37). The increase of Se in the diet reduced the influence of heat stress on the integrity of the intestinal barrier by reducing oxidative stress products (38). Subsequent experiment results also revealed that there was few ROS in the intestine, the reduction of tight junction protein expression increased the paracellular permeability, which was conducive to the absorption of nutrients.

### Goblet Cells and Goblet Cell Differentiation Regulator

The intestinal mucus layer is one of the main mechanical barriers with an inner, attached mucus and an outer, unattached, loose mucus layer (39). The major building block in mucus is Muc2, which is mainly synthesized and secreted by goblet cells (40). The destruction of the mucus barrier by pathogenic bacteria may make the goblet cells compensatorily increase the synthesis and secretion of Muc2 to expel the invasion of pathogenic bacteria (41). Moreover, goblet cells could protect the intestinal crypts from the invasion of bacteria, which had penetrated the inner mucus layer (12). Selenium deficiency during the growth of the broiler could increase cytoplasm vacuolization and dissolution of goblet cells (42). It was reported that SeNPs could increase the number of goblet cells and improve the structure of mucin in the mouse jejunum (14, 18). Khajeh Bami et al. (29) also found that broilers fed 0.3 mg/kg SeNPs had higher goblet cell density in the ileum and jejunum. Similarly, the results of this experiment showed that the number of goblet cells and the mRNA expression of *Muc2* in the jejunum were significantly increased by adding 0.1 mg/kg SeNPs.

Klf4 is a zinc finger transcription factor that is expressed in intestinal epithelial villi cells and can promote cell differentiation and tissue homeostasis (43). C-myc is a critical downstream effector of cellular proliferation induced by the Wnt/β-catenin pathway (44), which is essential for maintaining intestinal homeostasis and regeneration (45). Nrf2 is a master regulator of cellular oxidative levels against environmental stresses, which regulates intestinal cell differentiation by the KEAP1-Nrf2 pathway (46). SeNPs could alleviate intestinal epithelial barrier dysfunction caused by oxidative stress *via* Nrf2 signaling pathway (47). At present, there are few references on the regulation and control of SeNPs on the development of goblet cells in the intestine of broilers. This experiment found that the mRNA expression of *Klf4, c-myc*, and *Nrf2* in the jejunum decreased significantly after adding 0.4 mg/kg SeNPs. These results demonstrated that low-dose SeNPs could effectively increase the number of goblet cells, and high-dose SeNPs might be toxic to intestinal cells and inhibited the differentiation of intestinal goblet cells.

### Intestinal Inflammation and Antioxidant Activity

ROS are produced by intestinal epithelial cells through oxygen metabolism and by enteric commensal bacteria (48, 49). When the production of ROS exceeds the antioxidant capacity of cells, oxidative stress occurs and causes oxidative damage to tissues and cells, even causing bodily disorders (50, 51). Selenium can activate the GPX and TrxR through the formation of Sec in biological systems, which makes them suitable for oxidation/reduction reactions (1, 2, 52). SeNPs have been shown to have an effect on intestinal histology by high antioxidant capacity and free radical scavenging efficiency, which can protect intestinal tissues from oxidative damage (32). Selenium deficiency can induce villi cell apoptosis via an oxidative stress-induced mitochondrial apoptosis pathway and decreased the antioxidant capacity in chickens (15, 42). The secretion of mucin from intestinal goblet cells depends on the production of ROS (11). Salmonella infection activated the Notch signaling pathway through ROS in the intestine, leading to the loss of goblet cells (53). The current study found that the concentration of ROS in the jejunum decreased significantly with the increase of SeNPs concentration.

The pro-inflammatory cytokines of the avian intestinal mucosa play a key role in the host response to infectious pathogens (54). IL-1β is a pro-inflammatory cytokine that regulates antibody and cell-mediated immune responses (55). IL-8 is a CXC chemokine that attracts leukocytes (mainly neutrophils) to the sites of inflammation (54). Selenium deficiency induced chicken intestinal villi cell apoptosis by an inflammatory signaling-induced death receptor pathway and attenuated chicken intestinal mucosal immunity via activation of NF-κB signaling pathway regulated by redox activity (15, 42). Studies showed that *in ovo* injection with Se could increase intestinal levels of gene transcripts encoding IL-1β, IL-6, and IL-8 to enhance immune responses against avian necrotic enteritis (56, 57). Since this experiment did not establish the oxidative damage model, this study showed that the mRNA expression of *IL-1*β in the jejunum decreased significantly with the increase of SeNPs concentration, and diet supplemented with higher doses of SeNPs did not promote the expression of *IL-8*. These results demonstrated that exogenous supplementation with SeNPs effectively reduced levels of oxidative stress and inflammation in the intestine.

### Pyroptosis

Cell death is an important process in development, homeostasis, and immune regulation of multicellular organisms (58). Overproduction of ROS, pathogen, and host molecules, can lead to cellar dysfunction, which further initiates inflammation and cell death (58, 59). This study, by the TUNEL method, was the first to show that TUNEL positive cells in the 0.2 and 0.4 mg/kg SeNPs groups were significantly lower than the control group. Similarly, the mRNA expression of *Bax* and *Bcl-2* in the jejunum decreased significantly with the increasing dose of SeNPs. In contrast, Se deficiency induced duodenal villi cell apoptosis and injury of the mucosal physical barrier of the small intestine (15, 42). The NLRP3/caspase-1/IL-1β pathway is considered to be main active pathway of inflammation, which can be triggered by increased ROS and pathogens in order to protect against infections (58). When the pathway is activated, NLRP3 and ASC combine to each other to activate caspase-1, resulting in IL-1β-driven inflammation (58, 60). The secretion of mucin from intestinal goblet cells depends on the assembly and activation of inflammasomes (11). Among them, NLRP6 can regulate the secretion of Muc2 in goblet cells and promote the formation of mucus layers (61). The intestinal inflammation activated the NLRP6 in the goblet cells to regulate the secretion of Muc2 and promote the formation of mucus layers (62). Goblet cells nonspecifically endocytosed and reacted to the TLR ligands by activating the NLRP6 downstream of TLR- and MyD88-dependent ROS synthesis (12). Moreover, NLRP3 could alleviate the oxidative stress and inflammation of chicken kidneys, and participate in the antagonistic effect of Se on inflammation caused by lead (63). There were few reports on the role of inflammasome in regulating intestinal cells of broilers. It was showed that the expression levels of *NLRP3, Casepase-1, IL-18, TLR2*, and *MyD88* in the jejunum decreased linearly with the increase of SeNPs concentration. Similarly, Selenomethionine reduced lipopolysaccharide-induced inflammation by inhibiting NLRP3 signaling pathway in chicken liver (64). Selenium also alleviated Pb-induced oxidative stress and inflammation by inhibiting the TLR - NLRP3 signaling pathway in chicken testes (65). Taken together, it is suggested that SeNPs reduced intestinal oxidative stress and inflammation by NLRP3/caspase-1/IL-1β signaling pathway.

## Conclusion

In conclusion, exogenous supplementation with 0.2 mg/kg SeNPs can decrease intestinal oxidative stress and inflammation by modifying the activation of NLRP3 signaling pathway in chicken intestine, thereby effectively promoting goblet cells development of 21-day-old broilers.

## Data Availability Statement

The original contributions presented in the study are included in the article/supplementary material, further inquiries can be directed to the corresponding authors.

## Ethics Statement

The animal study was reviewed and approved by the Animal Ethics Committee of the China Agricultural University.

## Author Contributions

YW and JY designed the research. YC performed the experiments, wrote the manuscript, and analyzed the data. XL and SL performed the experiments. WW and HZ participated in the revision of the manuscript. YG and YL provided experimental guidance and experimental materials. All authors contributed to data interpretation and approved the final version of the article.

## Funding

This research was supported by National Key R&D Program of China (2021YFD1300404).

## Conflict of Interest

YL was employed by Beijing Wahmix Bio-technology Co., Ltd. The remaining authors declare that the research was conducted in the absence of any commercial or financial relationships that could be construed as a potential conflict of interest.

## Publisher's Note

All claims expressed in this article are solely those of the authors and do not necessarily represent those of their affiliated organizations, or those of the publisher, the editors and the reviewers. Any product that may be evaluated in this article, or claim that may be made by its manufacturer, is not guaranteed or endorsed by the publisher.
